# Micro and nanotechnologies: The little formulations that could

**DOI:** 10.1002/btm2.10421

**Published:** 2022-10-18

**Authors:** Rebeca T. Stiepel, Eliza Duggan, Cole J. Batty, Kristy M. Ainslie

**Affiliations:** ^1^ Division of Pharmacoengineering and Molecular Pharmaceutics, Eshelman School of Pharmacy University of North Carolina Chapel Hill North Carolina USA; ^2^ North Carolina School of Science and Mathematics Durham North Carolina USA; ^3^ Joint Department of Biomedical Engineering University of North Carolina at Chapel Hill and North Carolina State University Chapel Hill North Carolina USA; ^4^ Department of Microbiology and Immunology, UNC School of Medicine University of North Carolina Chapel Hill North Carolina USA

**Keywords:** inorganic, imaging agents, lipid nanoparticles, nanoparticles, PEG, vaccines

## Abstract

The first publication of micro‐ and nanotechnology in medicine was in 1798 with the use of the Cowpox virus by Edward Jenner as an attenuated vaccine against Smallpox. Since then, there has been an explosion of micro‐ and nanotechnologies for medical applications. The breadth of these micro‐ and nanotechnologies is discussed in this piece, presenting the date of their first report and their latest progression (e.g., clinical trials, FDA approval). This includes successes such as the recent severe acute respiratory syndrome coronavirus 2 (SARS‐CoV‐2) vaccines from Pfizer, Moderna, and Janssen (Johnson & Johnson) as well as the most popular nanoparticle therapy, liposomal Doxil. However, the enormity of the success of these platforms has not been without challenges. For example, we discuss why the production of Doxil was halted for several years, and the bankruptcy of BIND therapeutics, which relied on a nanoparticle drug carrier. Overall, the field of micro‐ and nanotechnology has advanced beyond these challenges and continues advancing new and novel platforms that have transformed therapies, vaccines, and imaging. In this review, a wide range of biomedical micro‐ and nanotechnology is discussed to serve as a primer to the field and provide an accessible summary of clinically relevant micro‐ and nanotechnology platforms.

## 1 DEFINING MICRO‐ AND NANOTECHNOLOGY

**FIGURE 1:**
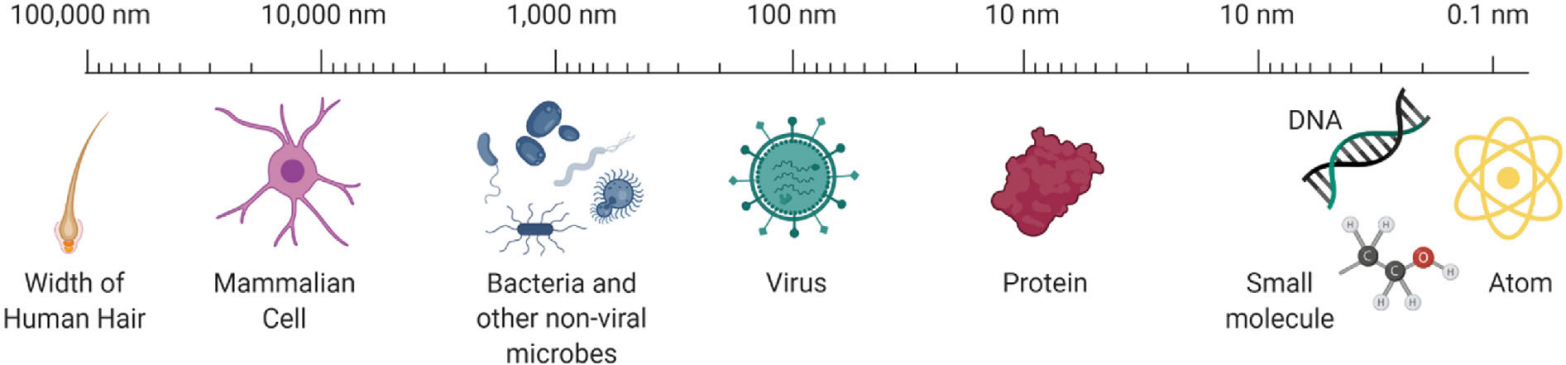
Scale of biological world. DNA, deoxyribonucleic acid; nm, nanometer

**FIGURE 2:**
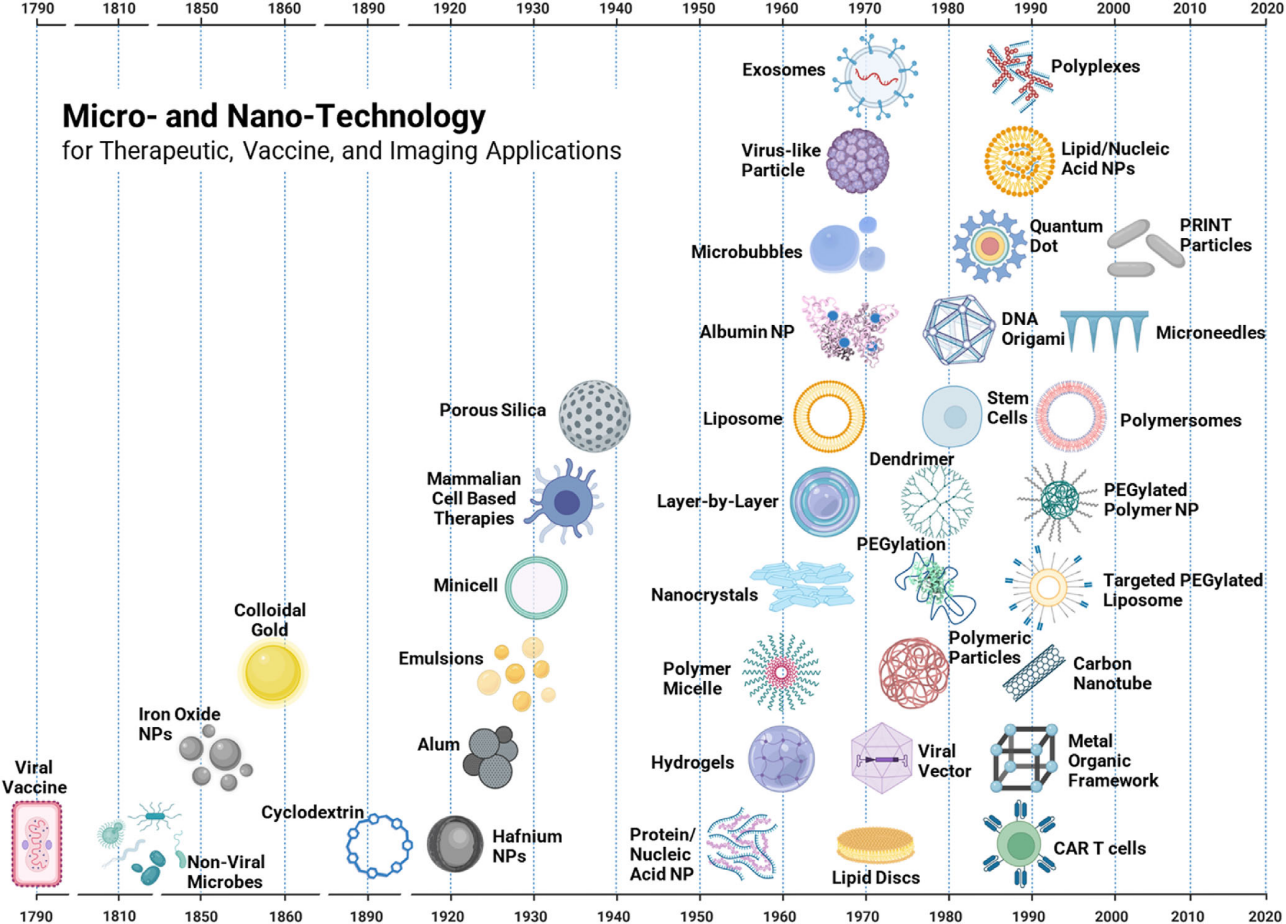
Timeline of first reports of micro and nanotechnology used for therapeutic, vaccine, and imaging applications. CAR T cells, chimeric antigen receptor T cells; NP, nanoparticle; PEG, polyethylene glycol; PEGylation/PEGylated, PEG covalently bound; PRINT, particle replication in nonwetting templates

Although the concept of nanotechnology was first introduced by Richard Feynman in 1959^1^, ^2^ and the term defined by Norio Taniguchi in 1974,^2^, ^3^ organic and inorganic materials that can be characterized as nanotechnology already existed. This is because the definition is based on size—they are simply technological products that have nanometer (nm) dimensions, and although they are applied in a variety of scientific and technical fields, this review will be highlighting nanotechnology through a biomedical lens. By National Institutes of Health (NIH) standards, nanotechnology ranges between 1 and 100 nm in size.^4^ By this strict definition, there are organic components common to the biological world (Figure 1) that fit within the scope of nanotechnology, such as DNA, protein, and viruses. These nano‐scale biological components have a longstanding history of clinical benefit. One very impactful example was Edward Jenner's development of Cowpox virus as a Smallpox vaccine in 1798^5^, ^6^ (Figure 2). This vaccine alone saved countless lives and facilitated the global eradication of the deadly disease Smallpox.^7^ The use of nanotechnology to eradicate a deadly disease underscores the impact of nanotechnologies and their influence in our world, even before they were given a definition.

Beyond nanotechnology and its historical definition, the range of these biomedical platforms has been tremendous (Figure 2; Table 1). As the concepts surrounding these platforms have grown, the definition of nanotechnology has often expanded to include elements ranging above 100 nm. As an example, several clinically approved formulations often considered to be nanoparticles^32^ exceed the NIH's size range for nano: Abraxane (130 nm),^130^ Inflexal V (150 nm),^131^ Myoset (150–250 nm),^130^, ^132^ TargomiRs (100–400 nm),^133^ SonoVue® (1610 nm),^134^ and Optison (3000–4500 nm).^135^ Additionally, several technological advances in nanomedicine include emerging biologics which are cellular in size (1–25+ μm). Therefore, to be inclusive of these platforms, the label micro‐ and nano‐technology can be used.

**TABLE 1:** First reports for micro‐ and nanotechnologies

		First report of advancement to the clinic or FDA approval			
Technology	First report	Status	Year	Product name	Indication
Viral vaccine A vaccine comprised of a virus^8^	1798^5^, ^6^	Used in patients	Predates FDA^a^	Cowpox virus	Smallpox
Nonviral microbes Bacteria, yeast, algae, and other nonviral microbes are used intact or in part for delivery or production of therapies^9^	1813^10^	Clinical trial NCT00922324	2009^11^	STP‐206	Necrotizing enterocolitis
Iron oxide NPs Comprised of iron and oxygen with magnetic and semiconductor properties^12^	1852^13^	FDA approved	1992^14^, ^a^	Feridex	MRI imaging
Colloidal gold A suspension of gold NPs dispersed in a fluid, typically water^15^	1857^16^, ^17^	Clinical trial NCT00356980	2006^18^	CYT‐6091	Solid tumors
Cyclodextrin Cyclic sugars produced from starch which can form toroid NPs^19^	1891^19^	FDA GRAS	1992^20^	Beta‐cyclodextrin	Food additive and pharmaceutical excipient
Hafnium oxide NP Particles made from hafnium^21^	1922^22^	Clinical trial NCT02805894	2016^23^	NBTXR3	Prostate adenocarcinoma
Alum Micron sized aluminum salts to which protein‐based antigens are adsorbed^24^, ^25^	1926^26^	FDA approved	1932^27^	Diphtheria toxoid with alum	Diphtheria vaccine
Emulsions A mixture of one liquid in another, usually oil in water^28^	1928^29^	FDA approved	1975^30^	Intralipid	For caloric intake
Minicell Small bacteria cells expelled from bacteria that cannot reproduce^31^	1930^31^	Clinical trial NCT02369198	2015^32^, ^33^	TargomiRs	Mesothelioma and lung cancer
Cell based therapy Host or other mammalian cells to deliver therapy^34^	1931^35^	FDA approved	2010^36^	Provenge	Prostate cancer
Porous silica Silicon dioxide particles with a high degree of porosity^37^	1939^38^	Clinical trial NCT01266096	2010^39^	C Dot	Melanoma
Protein/nucleic acid NP Positively charged proteins or peptides ionically complexed with negatively charged nucleic acids^40^	1955^41^	Clinical trial NCT03713086	2018^42^	CV7202	Rabies vaccine
Hydrogels Crosslinked hydrophilic polymer networks which typically swell in water^43^	1960^44^	FDA approved	1971^45^	Soflens	Contact lenses to correct vision
Polymer micelle Amphiphilic polymers which self‐assemble into a typically spherical aggregate^46^	1960^47^	Clinical trial NCT00111904	2005^48^	Genexol‐PM	Pancreatic cancer
Nanocrystals Nano‐sized crystals of a poorly soluble drug stabilized with surfactant or polymer coatings^49^	1961^50^	FDA approved	2003^51^	Emend	Antiemetic
Liposome Amphiphilic lipid bilayer vesicles^52^	1964^53^	FDA approved	1995^54^	Doxil	Kaposi's sarcoma
Layer‐by‐layer A charged core is layered with alternatively charged polymers, materials, or cargo^55^	1966^56^	Preclinical studies			Drug & gene delivery,^57^ vaccines & immunotherapy,^58^ imaging^59^
Albumin NP Albumin with drug adsorbed in hydrophobic regions of the protein^60^	1967^61^	FDA approved	2005^62^	Abraxane	Pancreatic cancer
Microbubbles Gas bubbles stabilized with a shell made from lipids, polymers or proteins^63^	1968^64^	FDA approved	1997^63^	Optison	Contrast agent
Virus‐like particle Self‐assembled viral proteins that form an empty virus sized sphere^65^	1968^66^	FDA approved	1989^67^, ^68^	Engerix‐B	Hepatitis B vaccine
Exosomes Cell secreted membrane bound vesicles with contents that can include nucleic acids, proteins, and lipids^69^	1971^70^	Clinical trial NCT00006430	2000^71^, ^72^	Dexosomes	Melanoma
Lipid discs Flat, circular lipid bilayer with a curved rim^73^	1971^74^	FDA approved	1996^75^, ^76^	Amphotec	Fungal infection
Viral vector Virus used to deliver nucleic acids^77^	1972^78^	FDA approved	2015^79^, ^80^	T‐VEC	Melanoma
Polymeric particles Spherical particles comprised of degradable or nondegradable polymers^81^	1976^82^	FDA approved	1989^83^, ^a^	Lupron depot	Endometriosis
PEGylation The covalent attachment of polyethylene glycol (PEG)^84^	1977^85^	FDA approved	1990^86^, ^a^	Adagen	PEG‐adenosine deaminase
Dendrimer Branching polymer NPs^87^	1978^88^	Clinical trial NCT00331032	2006^89^	SPL7013	Anti‐viral vaginal gel
Stem cells A cell that can develop into multiple cell types^90^	1981^91^, ^92^	FDA approved	2011^93^	Hemacord	Aberrant Hematopoiesis
DNA origami 3D structures of DNA^94^	1982^94^	Preclinical			Drug & gene delivery, vaccines & immunotherapy, imaging^95^, ^96^
Quantum dot Semiconductor crystals nm in size^97^	1985^98^	Preclinical			Imaging, drug delivery, theranostic^99^, ^100^
Lipid/nucleic acid NP Ionizable, charged and/or neutral lipids ionically assembled with negatively charged nucleic acids^101^	1987^102^	FDA approved	2018^103^	Onpattro	Amyloidosis
Polyplexes Positively charged polymers ionically complexed with negatively charged nucleic acids^104^	1987^105^	Clinical trial NCT00711997 NCT00393809	2006^106^	DTA‐H19	Bladder cancer
CAR T cells T cells genetically engineered to lyse cells which display specific epitopes^107^	1989^107^	FDA approved	2017^108^	Kymriah, CTL019	Acute lymphoblastic leukemia (ALL)
Metal organic framework Organic and metal ordered porous solids^109^	1989^110^	Clinical trial NCT03444714	2018^111^	RiMO‐301	Tumor radiotherapy
Carbon nanotube Hollow tube of carbon atoms^112^	1991^113^	Preclinical			Drug delivery, vaccines, imaging, theranostics^114^, ^115^
Targeted PEGylated liposome Liposome with PEG outer layer and active targeting moieties^116^	1992^117^	Preclinical			Drug delivery^116^
PEGylated polymer NP Polymeric NPs with covalent attachment of PEG^118^	1994^119^	Clinical trial NCT01300533	2011^120^	BIND‐014	Cancer
Polymersomes Self‐assembled block copolymer bilayer vesicles^121^	1995^122^, ^123^	Preclinical			Drug delivery^124^
Microneedles Array of micron‐scaled needles for transdermal delivery^125^	1998^125^	Clinical trial	2001^126^	Microneedles	Pain with application
PRINT particles Molded particles comprised commonly of polymers^127^	2005^128^	Clinical trial NCT01224262	2010^129^	LIQ001	Influenza vaccine

Abbreviations: CAR, chimeric antigen receptor; FDA, US Food and Drug Administration; MRI, magnetic resonance imaging; NCT, national clinical trial, which is followed by the number for that trial; NP, nanoparticle; PEG, polyethylene glycol; PRINT, particle replication in nonwetting templates.

^a^ Product was discontinued.

This review focuses on why biomedical technologies in this size range are unique, as well as the highs and lows of these technologies in medical applications. To give the breadth of successes of these platforms, a timeline of discovery of the most referenced micro‐ and nanotechnologies is illustrated and presented with a short description and information related to their discovery, clinical trials, and United States (US) Food and Drug Administration (FDA) approval. Referencing available literature regarding the challenges of the development of these platforms is also included to highlight the obstacles the field has encountered. Technologies in preclinical and clinical application are also included, noting that a review of FDA approved nanoparticles is provided by Anselmo et al.^32^

## 2 WHAT MAKES MICRO‐ AND NANOTECHNOLOGY PLATFORMS SO SPECIAL?

A wide range of micro‐ and nanotechnology platforms have been applied preclinically and clinically to improve the delivery of therapeutics, vaccines, and imaging agents (Figure 2). Micro‐ and nanomaterials can have unique properties due to the number of atoms they have on their surface, compared to their volume—they possess a higher surface area to volume ratio. For example, if a typical carbon–carbon bond is approximately 0.154 nm in length,^136^ a 1 nm diameter sphere has approximately 61.6% of its atoms on the surface while a 1 cm diameter sphere has approximately 6.16 × 10^−6^% of its atoms on the surface (Table 2). Surface atoms have a greater opportunity to receive energy from the environment in the forms of heat, light, and sound, which can change their physiochemical behavior in comparison to bulk materials. Additionally, a higher percentage of atoms on the surface allows for a higher fraction of the material's overall volume to interact with cells, proteins, and other constituents of biological systems, leading to unique biological interactions.

**TABLE 2:** Estimated percent of atoms on the surface of nanometer to meter scaled materials assuming a sphere packing density of ~74%

Sphere diameter	Volume (nm^3^)	Approximate total number of carbon atoms	Approximate number of carbon atoms on surface	Percent of atoms on surface (%)
1 nm	5.24e−01	203	125	61.6
1 μm	5.24e+08	2.03e+11	1.25e+08	6.16e−02
1 cm	5.24e+20	2.03e+23	1.25e+16	6.16e−06
1 m	5.24e+26	2.03e+29	1.25e+20	6.16e−08

In addition to having a higher surface area to volume ratio, the smaller scale of micro‐ and nanomaterials allows for specific interactions with individual cells (Figure 1). The scale of biomedical material platforms is critical because even something as small as 1 cm is 1000 times (or more) greater than the size of a mammalian cell. In applications where interactions between individual cells and a biomaterial platform are essential to the intended effect, this purpose cannot be meaningfully fulfilled when the material is orders of magnitude larger than the cell. Nevertheless, for biological applications before micro‐ and nanotechnology platforms were regularly applied, formulations were 1000 times or more the size of cells and proteins. By reducing the size of biomaterials to the micro‐ and nano‐scale, crucial interactions between these materials and structures in the body can be more easily facilitated. Additional material properties such as charge density and surface chemistry as well as chosen systemic or local routes of administration (e.g., oral, rectal, intravenous, subcutaneous, intramuscular, intranasal) can be optimized to best facilitate desired interactions between cells and micro‐ and nanotechnology platforms.^6^

## 3 CLASSES OF THERAPEUTIC, VACCINE, AND IMAGING TECHNOLOGIES

There are several classes of biomedical nanotechnologies that can be identified. Most are simply classified as inorganic or organic carriers. However, organic carriers can be further classified into technologies comprised of polymers, lipids, or nonlipid biologics. Additionally, combinations of one or more inorganic or organic materials have been used. Below we discuss technologies incorporating these various materials, with the understanding that many can carry cargo that differs from what the vehicle itself is comprised of. These cargoes can include peptides, proteins, nucleic acids, and small molecules. Of note, this section highlights classes of biomedical carriers to give an overview of the platforms. Further discussion of successes and challenges can be found in later sections.

### 3.1 Inorganic

Inorganic nanotechnology platforms were described in literature prior to the definition of the term. Of all the inorganic materials that occur naturally or synthetically, metals are most commonly applied in micro‐ and nanotechnology because they make excellent imaging agents.^137^ Further, the imaging properties of these inorganic platforms are sometimes tied with delivery of therapeutic agents to form theranostics—platforms which are both therapeutic and diagnostic. Magnetite (iron oxide) nanoparticles (NPs) were first synthesized in 1852 by Lefort and later applied as imaging and drug delivery technologies including the 1992 FDA approved Feridex NP.^13^, ^14^ Further, gold NPs were first formed by Faraday in 1857, and applied for similar imaging and drug delivery applications.^16^ Also used for imaging are hafnium nanoparticles which were isolated from zirconium compounds in 1922^22^ and have advanced to Clinical Trials (National Clinical Trial [NCT] Number 02805894). Additionally, quantum dots (Q dots) are an emerging inorganic NP for medical imaging which is comprised of semiconductor materials and emits fluorescent signal. Q dots were discovered in 1985^98^ and are currently being evaluated preclinically for applications in nuclear medicine where they have potential to be used for imaging and diagnostics.^138^, ^139^ These aforementioned inorganic platforms possess a wide variety of clinical applications, including tumor imaging with the goal of facilitating innumerable life‐saving treatments for cancer.

Three additional inorganic micro‐ and nanotechnology platforms are alum, porous silica, and carbon nanotubes. Alum is a microstructure of an aluminum salt, whose adjuvant activity was discovered in 1926^26^ when protein antigens were adsorbed to its surface and used for vaccination. In 1932^27^ it was applied as a Diphtheria vaccine in humans. Porous silica was first discovered in 1939^38^ and has advanced to clinical trials with the Cornell Dot (C DOT) entering the first trial in 2010^39^ for imaging of melanoma and other tumors. Besides imaging applications, the high surface area of porous silica can allow loading of therapeutics through covalent attachment or adsorption, allowing for use as a vaccine or drug carrier. More recently, carbon nanotubes were reported in 1991^113^ and have been applied preclinically for applications in imaging, drug delivery, and vaccines.^140^, ^141^

### 3.2 Polymer based

There are several micro and nanotechnologies comprised mostly of polymers (Table 2). In 1960, an early report on polymeric technologies focused on hydrogels.^44^ Hydrogels are made of crosslinked‐hydrophilic polymers that traditionally swell in the presence of water, making them ideal for use in soft‐contact lenses, for which they were first approved in 1971.^45^ Hydrogels are also used as drug delivery systems to achieve a controlled release of therapeutic cargo over time.

**FIGURE 3:**
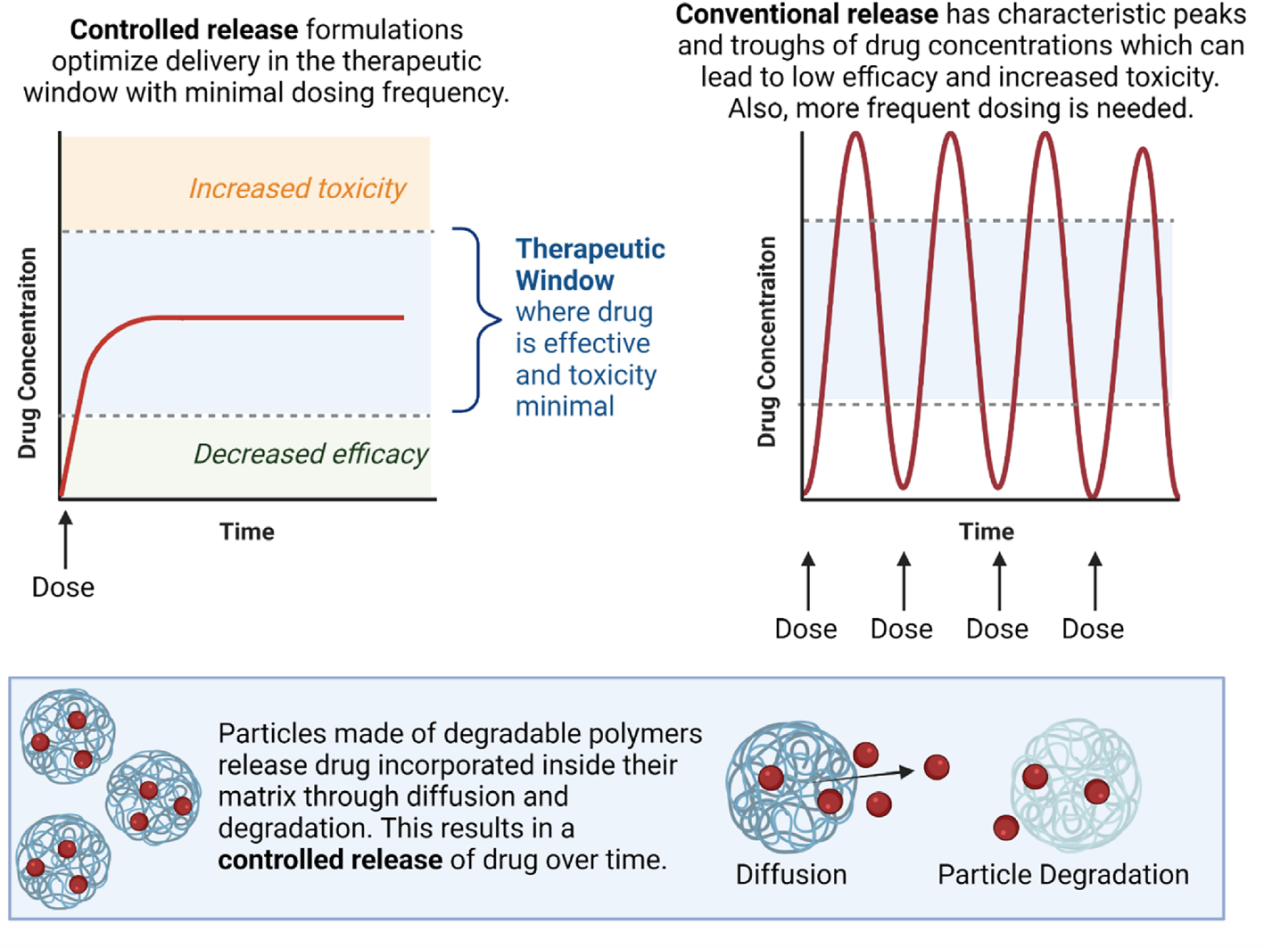
Controlled release defined and illustrated with release of drug from polymeric particles

Additional drug delivery platforms are often comprised of polymeric particles, which are often created by emulsion (e.g., homogenization, sonication) or spray (e.g., electrospray, spray drying) methods. Both types of methods can successfully form polymeric particles encapsulating a variety of cargos. Although, high speed shearing associated with emulsion methods can negatively affect protein antigens,^142^ and there are some limitations of solvent selection with spray methods.^143^, ^144^ The best fabrication method can vary by cargo and application of the particle system. These systems typically employ a hydrophobic polymer, which does not need to be crosslinked to prevent dissolution in vivo. These polymeric particles were first reported in 1976^82^ and FDA approved under the name Lupron Depot in 1989^83^ for treatment of endometriosis. Both hydrogels and polymeric particles can be classified as controlled release systems and often employ degradable polymers to facilitate the release of therapeutic encapsulates (Figure 3). These controlled release systems are valuable for maintaining therapeutic dosing at the intended site of drug delivery while reducing dosing frequency and minimizing toxic off target effects.

Polymer‐based micro and nano‐structures can also be fabricated by micromolding, a technique wherein a master structure is created through microfabrication, usually from silica. A mold is then made from the master, typically out of polydimethylsiloxane (PDMS). Another polymer solution is poured into the mold and left to harden. Once removed, the hardened polymer is in the shape of the master.^145^ Two examples of micromolding are microneedles and particle replication in nonwetting templates (PRINT) particles. Microneedles were originally reported in 1998^125^ and comprised of inorganic materials.^145^ Eventually, the inorganic materials were used as masters for the fabrication of polymeric microneedles.^146^ In 2001, the first clinical trial for microneedles was conducted to evaluate pain upon application, validating the platform as a painless transdermal drug delivery system.^126^ In the case of PRINT particles, micromolding techniques were modified to use a highly fluoridated polymer to create a mold that resists wetting. With this modification, nano and micro‐scaled particles have been made. These PRINT particles were first reported in 2005^128^ and advanced to clinical trials as an influenza vaccine adjuvant in 2010.^129^

**FIGURE 4:**
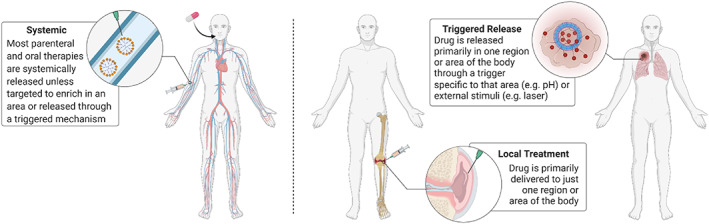
Graphic depicting administration route which compares systemic administration versus triggered release or local treatment. Triggered release or local treatment can concentrate a drug at one region or area of the body to mitigate potential off‐target toxicities.

The ability to tune polymer properties through monomer selection and copolymerization makes this material class highly tunable for biomedical applications. One example of this is the generation of amphiphilic macromolecules that exhibit both hydrophilic and lipophilic properties by forming a copolymer of a hydrophilic monomer/polymer (e.g., polyethylene glycol (PEG)) and a lipophilic monomer/polymer (e.g., polyesters like poly[lactic acid]). In 1960,^47^ amphiphilic polymers were used to construct polymer micelles that can encapsulate hydrophobic cargo such as the chemotherapeutic paclitaxel. Paclitaxel loaded polymer micelles under the name of Genexol‐PM reached clinical trials to treat pancreatic cancer in 2005.^48^ When amphiphilic polymers are self‐assembled into a spherical bilayer, they form polymersomes. Polymersomes were first reported in 1995^122^, ^123^ and are under preclinical evaluation. Of interest, stimuli‐responsive polymersomes are under evaluation to achieve triggered release of therapeutics upon specific stimuli, including external physical manipulation (e.g., temperature, light, electric field, ultrasound) or specific internal biologic factors (e.g., pH).^147^ The triggered release achieved by stimuli‐responsive materials, including polymersomes, has potential for clinical benefit in cases where the drug is more localized to the site of interest (e.g., tumor), which can minimize off‐target toxicities (Figure 4).

Two additional strategies to tune polymer properties include the addition of chemical handles to covalently attach cargo or the inclusion of desired chemical groups to instill charge in the NP. These strategies are exemplified in dendrimers, polyplexes, and layer‐by‐layer particles. Initially developed in 1978,^88^ dendrimers are branching polymers wherein a chemical core is created and subsequent reactions are carried out for each generation of branching. Since each generation has specific chemistries, dendrimers can result in a three‐dimensional (3D) star‐like polymer with specific functional handles (e.g., amine, carboxylic acid) which can be used to attach various cargoes including proteins, peptides, small molecules, and/or targeting ligands. As an example, SPL7013 advanced to clinical trials in 2006^89^ as a locally delivered vaginal gel containing dendrimers with anti‐viral ligands. Additional drug carrier modifications can be made with monomer selection, where the charge of the polymer can be modified to ionically complex charged payloads. In the case of polyplexes, which were first reported in 1987,^105^ a cationic polymer is ionically complexed with negatively charged mRNA, DNA, or other nucleic acid. Once complexed, the charge of the nucleic acid can be reduced or neutralized, allowing for better uptake by the cell.^148^, ^149^ Polyplexes reached clinical trials in 2006^106^ with DTA‐H19 for the treatment of bladder cancer. Furthermore, more complex polyplexes can be made by a layer‐by‐layer method to form multiple layers of cationic and anionic polymers and/or nucleic acids. In these layer‐by‐layer polyplexes, a solid shell is often used initially with alternating charged polymers, nucleic acids, or additional cargoes added for each layer, often achieving unique release kinetics.^55^ This method was first reported in 1966^56^ and is under preclinical evaluation for applications in vaccines and delivery of therapeutic small molecules, proteins, and/or cells.^55^

**FIGURE 5:**
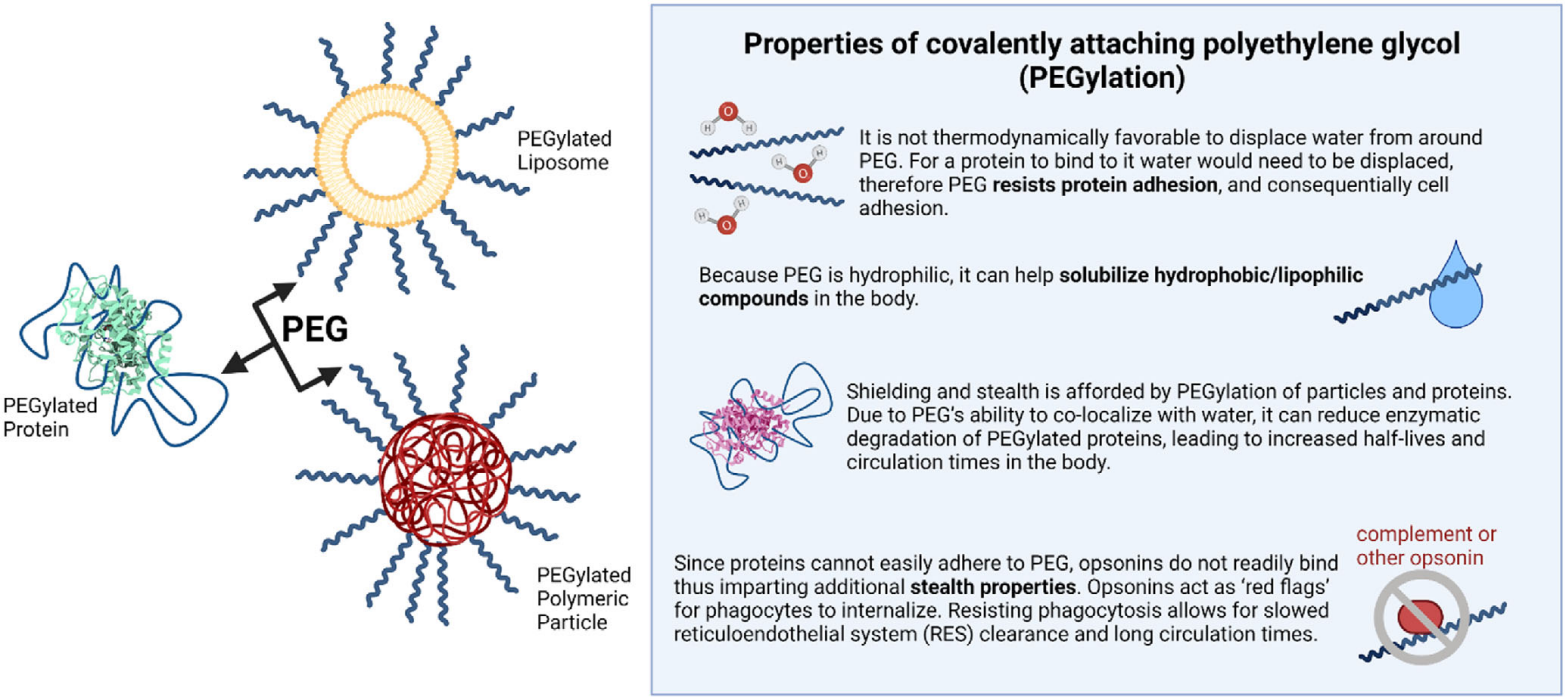
Graphic highlighting the properties that PEGylation can impart on micro‐ and nanotechnology‐based formulations.^150^, ^151^

A significant advancement in drug delivery was the covalent attachment of PEG to therapies and drug carriers (PEGylation). The addition of PEG can help solubilize lipophilic cargo in the body and increase circulation times because it limits protein adsorption due to the polymer's ability to colocalize water molecules (Figure 5).^152^ By inhibiting the binding of proteins, PEG restricts molecules that act as “red flags” (opsonins) to signal internalization and clearance by the reticuloendothelial system (RES). Additionally, PEG can protect the cargo from enzymatic degradation by steric interactions and/or the colocalization of water, thus also increasing the cargo's half‐life in the body. The effects of PEGylation can be further tuned with the density of PEG chains as well as their conformation. For instance, particles with dense PEG chains in a long “brush” morphology are less likely to be taken up by cells than particles with less dense PEG chains in a shorter “mushroom” morphology.^153^ First identified in 1977,^85^ PEGylated adenosine deaminase (Adagen) was the first PEGylated therapy approved in 1990,^86^ though it was later discontinued from the market due to insufficient supply of bovine adenosine deaminase.^154^ Due to the ubiquity of PEGylation applied to micro‐ and nanotechnology, further discussion on PEG applied to drug delivery platforms can be found in the following sections: “Success can Come in Small Packages” and “Challenges in the Field.”

**FIGURE 6:**
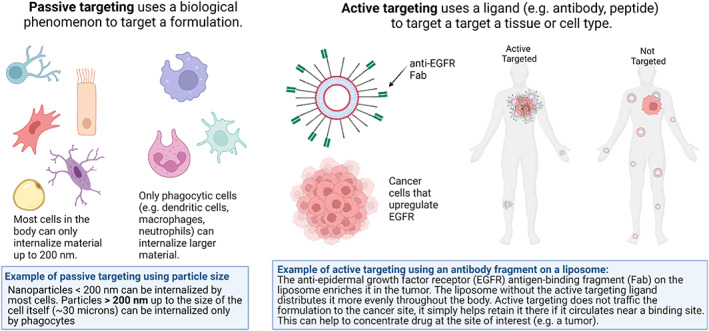
Graphic contrasting passive and active targeting with two examples. Passive targeting is exemplified by using size to exclude uptake in most cells, for preferential uptake in phagocytic cells. Active targeting is illustrated by using an antibody fragment against a receptor (EGFR) that is upregulated on cancer cells.^6^, ^155^

Although PEG has been used to functionalize many drug carriers, functionalization via the addition of a targeting group is a significant feature in micro‐ and nanotechnology. The addition of targeting most often refers to active targeting (Figure 6), which can allow enrichment of a drug or carrier in a site of interest, such as a tumor. By enriching the drug or carrier in an area of interest, the off‐target effects should be reduced while the amount of desired cargo at the region of interest is increased (e.g., tumor site). In 1992,^117^ the first study was reported wherein a PEGylated liposome was functionalized at the end of the PEG chain with a targeting ligand to achieve longer circulation times and increase delivery to necrotic sites after myocardial infarction. This work continues to be preclinical, primarily for the delivery of therapeutic agents for cancer.

### 3.3 Lipid based

Some of the most impactful micro‐ and nanotechnology carrier systems are lipid based. This includes the highly utilized liposome, the closely related lipid disc, and lipid and nucleic acid NPs (often referred to as LNPs, although strictly this definition includes all the aforementioned lipid particles)—which were utilized in the Pfizer and Moderna severe acute respiratory syndrome coronavirus 2 (SARS‐CoV‐2) vaccines to prevent development of the disease COVID‐19.^156^ A majority of lipid‐based carriers use phospholipids that are amphiphilic in nature and can self‐assemble into single layers (lipid micelles), bilayers (liposomes, discs), or aggregated single and multilayers (LNPs).^157^ Liposomes were the first reported lipid carrier in 1964^53^ and the first to reach FDA approval in 1995.^54^ Liposomes can be modified to make lipid discs. As such, once lipids are above the saturation point for the bilayer, curvature can occur spontaneously, particularly below the critical temperature of the lipid mixture.^158^ Lipid discs were first reported in 1971^74^ and approved under the name Amphotec in 1996 for delivery of amphotericin B to treat fungal infections.^75^, ^76^ Regarding LNPs, although they have gained recent prominence for their use in two of the COVID‐19 vaccines, they have been previously highlighted in literature since 1987^102^ and FDA approved in 2018^103^ for delivery of short‐interfering RNA (siRNA) for treatment of amyloidosis (Onpattro).

### 3.4 Combination technologies

Some carriers are comprised of a combination of organic and inorganic materials. One combination technology that has advanced to clinical trials is metal organic frameworks (MOFs). MOFs have metal ions (e.g., zinc) with organic linker molecules (e.g., imidazolate linkers) that form complex 3D structures which can encapsulate cargo or provide a high surface area for adsorption.^159^ MOFs were first reported in 1989,^110^ and they have already advanced to clinical trials in 2018^111^ for tumor radiotherapy.

### 3.5 Nonlipid biologics

With the inclusion of microtechnologies, the scale (Figure 1) moves beyond 100 nm to include nonviral microbes (e.g., bacteria, yeast, algae) and mammalian cells. Nonviral microbe‐based therapies were reported as early back as 1813 when the bacteria *Clostridium perfringens* was used to slow the growth of a tumor in a cancer patient.^9^ More recently bacteria as a platform has been used clinically for fecal microbiota transplants (FMTs) to treat *Clostridium difficile* infection,^160^ and FMTs are being evaluated for other conditions associated with microbiome dysbiosis, including but not limited to inflammatory bowel disease.^161^ In 2009,^11^ a whole cell bacteria therapeutic (STP‐206) entered clinical trials for necrotizing enterocolitis. Mammalian cell based therapies include cell based Provenge (approved in 2010)^36^ as a prostate cancer vaccine, stem cell therapies like Hemacord (approved in 2011)^93^ to treat patients who cannot produce their own blood cells, and the chimeric antigen receptor (CAR) T cell therapy Kymriah (approved in 2017)^108^ for the treatment of leukemia and lymphoma. These mammalian cell‐based therapies illustrate a more tailored approach that can require patient samples (e.g., blood, tumor) and perhaps genetic sequencing to develop a more personalized treatment. For example, in the generation of CAR T cells,^162^ T cells are purified from patient blood, activated, transfected, expanded, formulated, and injected back into the patient. This is in contrast to the aforementioned attenuated viral vaccine identified by Jenner, which was a one‐size‐fits‐all approach to immunomodulation.

Micro‐ and nanotechnologies can also be produced by cells. Two examples are minicells and exosomes. Minicells were first identified in 1930^31^ when buds were expelled from bacteria, but these buddings were not able to replicate. In 2015,^32^, ^33^ minicells containing a microRNA mimic (TargomIRs) were first reported in clinical trials for treatment of cancer. Similar to minicells, exosomes are membrane‐based vesicles that are expelled from mammalian cells. Exosomes were first identified in 1971^70^ and can contain a variety of nucleic acids, proteins, and lipids. Dexosomes were the first exosomes to enter clinical trials in 2000^71^, ^72^ for the treatment of melanoma.

In addition to cell‐related technologies, viruses are used for therapeutic and vaccine applications. As previously discussed, the earliest recorded use of this technology was Jenner's use of Cowpox virus in 1798^5^, ^6^ for vaccination against Smallpox. Jenner applied a naturally occurring virus as the vaccine, whereas modern viral vectors modify viruses to deliver specific nucleic acids. The first viral vector therapy was reported in 1972^78^ when Simian Virus 40 was modified. The first FDA approved viral vector was in 2015,^79^, ^80^ utilizing herpes simplex virus 1 (HSV‐1) to deliver immunomodulatory proteins to treat melanoma. Closely related to a virus but comprised of viral proteins that self‐assemble into a virus‐like sphere are virus‐like particles (VLPs). In contrast to Jenner's vaccine that used whole virus with capsid and DNA, a VLP contains no DNA or viral replicating machinery, but only contains a few select proteins that serve as vaccine antigens or structural elements to facilitate self‐assembly. VLPs were first reported in 1968^66^ and FDA approved for a Hepatitis B vaccine in 1989.^67^, ^68^

The last three biologic based micro and nanotechnologies discussed herein are comprised of proteins and/or nucleic acids. Similar to LNPs, protein and nucleic acid NPs use a cationic protein (e.g., protamine) to ionically complex negatively charged nucleic acids. These NPs were first reported in 1955^41^ and progressed to clinical trials in 2018^42^ as a rabies vaccine. Second, the abundant blood protein albumin can be used as a NP wherein the hydrophobic pockets of the protein are used to adsorb hydrophobic cargo. This was first reported in 1967^61^ and FDA approved as a pancreatic cancer treatment (Abraxane) in 2005.^62^ Third, a DNA based technology, DNA origami, was first reported in 1982.^94^ The specific hybridization of complementary DNA is used to create 3D structures out of DNA, which has been studied preclinically for biomedical applications such as drug and gene delivery and vaccines.

### 3.6 Other materials

There are several technologies that are comprised largely of other materials. An emulsion is a dispersion of one liquid in another liquid in which it is immiscible. Typically water and a food‐based oil (e.g., soybean) are used in the biomedical field. Biomedical emulsions were first reported in literature in 1928^29^ and approved by the FDA in 1975^30^ as Intralipid, which delivers a high mass fraction of fats to patients who need to increase their caloric intake. As another example, cyclodextrins are unique macromolecules comprised of cyclic sugars isolated from starch. They can stack and form doughnut shaped NPs. First reported in 1891,^19^ they are listed on the FDA Generally Regarded as Safe (GRAS) list because of their natural abundance in food. The GRAS list is comprised of chemicals that are approved by the FDA for use in food additives because they are considered safe within certain dose ranges. Cyclodextrin's inclusion on this list was noted in 1992,^20^ and they have since been applied as an intentional food additive and pharmaceutical excipient. As a carrier in biomedical applications, it can be used to adsorb or ligate cargo and has advanced to clinical trials.^32^ Another drug carrier, which is comprised mostly of drug with a small amount of excipient (~10% wt/wt), is a nanocrystal.^49^ Nanocrystals were first reported in 1961^50^ and FDA approved as an antiemetic in 2003.^51^ A final technology is a microbubble which consists of a gas bubble stabilized with lipids or proteins. These are most often used as contrast agents for imaging and were first reported in 1968.^64^ In 1997^63^ they were FDA approved as a contrast agent.

These aforementioned micro‐ and nano‐technologies illustrate a long history of accomplishments in the biomedical field for enhanced delivery of therapies, vaccines, and imaging agents. Several of these platforms are used for multiple applications and can deliver a variety of therapies making their use ubiquitous and their potential successes limitless.

## 4 SUCCESS CAN COME IN SMALL PACKAGES

Perhaps foreshadowed by Jenner's Smallpox vaccine which prevented substantial morbidity and mortality during a deadly pandemic, the most significant success of nanotechnology in the 21st century was the rapid production of lipid nanoparticles (LNPs) and adenoviruses for vaccination against SARS‐CoV2, the virus that causes COVID‐19.^156^ Using LNPs with ionizable lipids,^163^ both Moderna and Pfizer incorporated modified mRNA encoding the spike protein of SARS‐CoV‐2 to produce effective COVID‐19 vaccines.^156^ The LNP platform utilized by both companies allowed for protection and delivery of mRNA cargo which elicited the generation of protective immune responses. These vaccines were FDA approved for emergency use in 2020, and in 2021 Pfizer received full approval.^164^ In addition to LNPs, adenovirus vectors were developed to combat COVID‐19. Although viral vectors had been previously reported and FDA approved,^78^, ^79^, ^80^ the type of viral vector (adenovirus delivering DNA encoding an antigen) used in the Johnson and Johnson (Janssen) vaccine had not been previously FDA approved.^156^ Both the adenovirus platform and the LNP platforms illustrated effective vaccine responses in patients and continue to do so.^165^, ^166^, ^167^ Moreover, they underscore the utility of nanotechnologies and their ability to have flexible biomedical applications that can be applied for rapid development.^168^

Another significant technological advance is the addition of PEG to therapies and drug carriers, including the LNPs developed by Pfizer and Moderna as well as the first FDA approved liposome, Doxil. Additionally, over 20^152^, ^169^ PEGylated therapies are currently FDA approved. Just as a copolymer has the properties of each of its separate monomers, the addition of PEG to a therapy can make a hydrophobic compound more water soluble. PEG is so significantly water‐loving that it is thermodynamically unfavorable for water to disassociate from the polymer^170^ (Figure 5). Because water would need to be displaced for protein adhesion, and protein adhesion facilitates cell attachment, PEG imparts resistance to both protein and cell adhesion. Decreased protein binding also leads to the inhibition of binding of complement and other opsonins which results in longer circulation times and reduced clearance by the reticuloendothelial system (RES) for PEGylated therapies. To illustrate the impact of PEGylation, a random controlled trial comparing PEGylated liposomal doxorubicin (e.g., Doxil) to conventional doxorubicin for the treatment of multiple myeloma illustrated that treatment with PEGylated liposomal doxorubicin resulted in similar efficacy and cost, but the PEGylated liposome resulted in less toxicity and supportive care.^171^ Overall, the reduction of side effects while maintaining therapeutic efficacy indicates greater clinical benefit with the PEGylated liposome. Furthermore, other clinical trials illustrated a similar reduction in toxicity with the PEGylated treatment over conventional options for breast cancer,^172^, ^173^ Kaposi's sarcoma,^174^ and soft tissue sarcoma.^175^ Together, these clinical findings underscore the therapeutic benefit of PEGylation (Figure 5).^152^

Notably, the application of the many of the aforementioned technologies can positively impact patient outcomes in resource limited settings. Two examples are liposomal amphotericin B (AmBisome) for treatment of leishmaniasis and long‐acting antiretroviral therapy (ART) for treatment and prevention of HIV/AIDS. The neglected tropical disease leishmaniasis (caused by around 20 species of the genus *Leishmania*) is the second most common parasitic disease after malaria and affects close to 1 million people yearly.^176^ It is endemic in places like Bihar, the second poorest state in India.^177^, ^178^ Although there are several FDA approved and preclinical treatments for leishmaniasis,^176^ amphotericin B is the first‐line treatment in endemic areas.^179^ Amphotericin B is a highly lipophilic drug that can disrupt the parasite's membrane and incorporates well in the lipid layer of liposomes (as in AmBisome) as well as the lipid disc formulation Amphotec. Conventional amphotericin B (amphotericin B deoxycholate) is given daily or every other day for approximately 15 doses, whereas AmBisome can be given in a single dose with significantly fewer toxicity concerns because of the controlled release afforded by the liposomal carrier.^180^, ^181^ In these resource limited settings, each day of conventional treatment is a day off of work for the individual, and other costs such as travel, food, and lodging to receive care can be significant.^182^ Moreover, patients are more likely not to complete their treatment if it has a long duration, resulting in incomplete clearance of the parasite and emergence of drug resistance.^176^ Monthly injectables and the decreased dosing frequency achieved with AmBisome lead to increased compliance and decreased drug resistance as a result of the micro‐ and nanotechnologies used. Additionally, it reduces the financial burden of time off work and travel to clinics for care. Similar reduced dosing frequencies are observed with the application of long‐acting ART nanocrystals for prevention and treatment of HIV/AIDS. Long acting cabotegravir and rilpivirine have been developed as intramuscular injected nanocrystals for sustained delivery of drug lasting for a month or more.^183^ Although oral delivery is thought to be paramount in drug delivery, the rate of adherence to oral ART is approximately 70% independent of assessment method. This is concerning considering that virologic failure (the inability to maintain suppression of viral replication) and drug resistance significantly increase when adherence rates fall below 90%–95%.^184^ As such, longer acting formulations can have substantial clinical benefit such as better adherence and less virologic failure.

Although there are other examples, the ones given here clearly outline how micro‐ and nanotechnologies have significantly impacted human health in a positive manner. This can include rapid generation to meet the needs of a pandemic and eradication of diseases as well as reduced drug toxicity and dosing frequency to increase patient compliance and decrease drug resistance.

## 5 CHALLENGES IN THE FIELD

**FIGURE 7:**
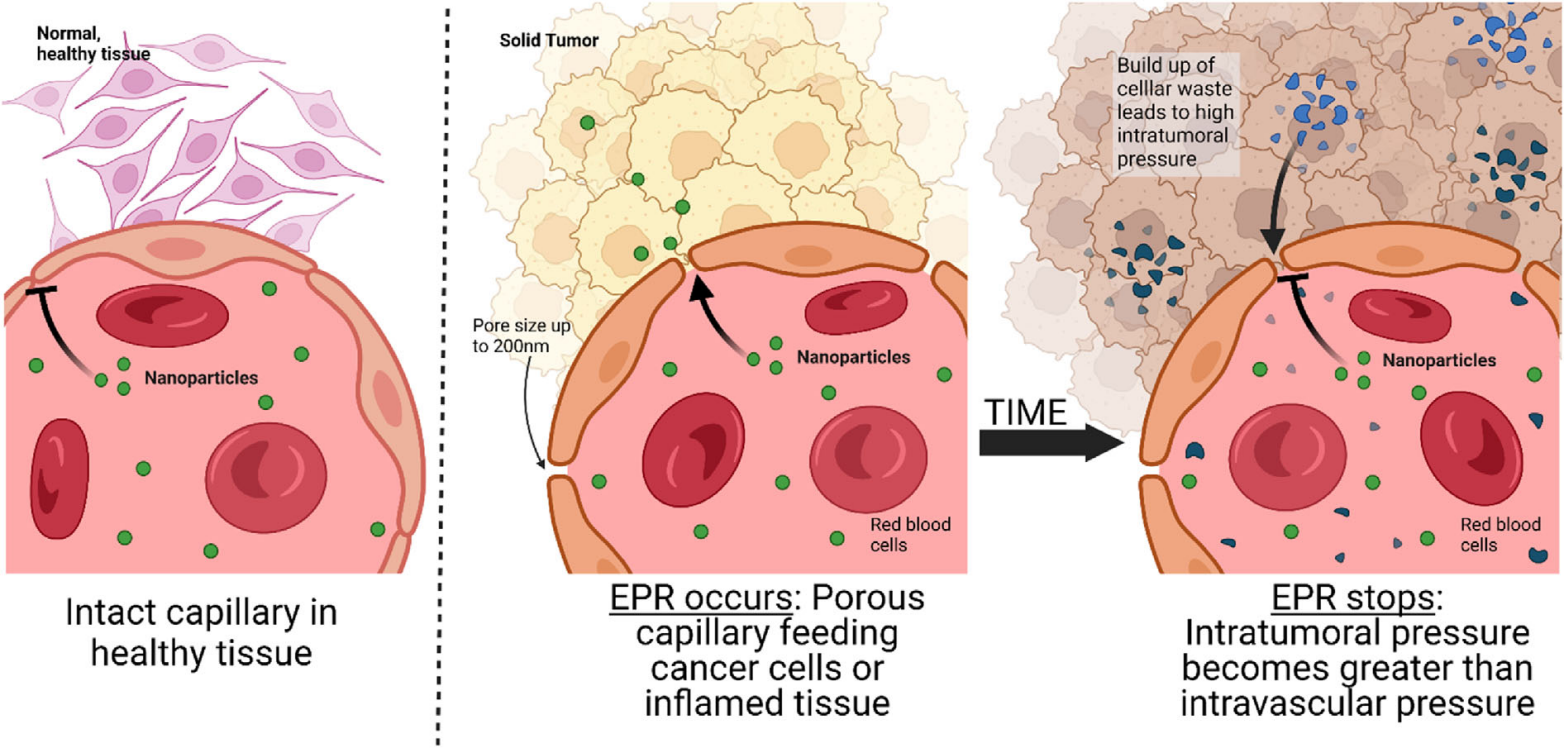
Illustration of passive targeting phenomena, the enhanced permeability and retention (EPR) effect. In intact capillaries, the NPs must transport between or through endothelial cells to traffic out of the vessel. In capillaries feeding inflamed and solid cancers, pores as large as 200 nm can form and allow NPs and other nano‐sized material in the blood to enter the surrounding tissue more easily. Over time, the lack of lymphatic drainage in solid tumors can result in buildup of material like cell waste, which results in a high intratumoral pressure. If the intratumoral pressure is greater than the pressure inside the capillary, then EPR no longer occurs.^185^

Since micro‐ and nanotechnology platforms have unique interactions with the biological world, a logical application would be to use them to better target cells and tissues of interest. These materials can be used to passively target areas of the body (Figure 6). For instance, micron sized particles can passively target phagocytic immune cells over nonphagocytic cells which typically only internalize material less than 200 nm (Figure 6).^6^ Most notably in drug delivery research, nanomaterials have been noted to accumulate in tumors and at sites of inflammation due to the enhanced permeability and retention (EPR) effect (Figure 7).^186^ The EPR effect occurs in solid tumors when the tumor reaches 1–2 centimeters in size, and can no longer feed all of its cells through diffusion from peripheral capillaries.^187^ The formation of capillaries by the cancer becomes haphazard and results in leaky junctions between endothelial cells and pores as large as 200 nm.^185^ However, there is controversy regarding actual accumulation of NPs in tumors due to EPR,^188^ despite evidence that the drug fraction in the tumor compared to the blood is improved with delivery via NPs.^189^ Some of this controversy can be attributed to observed differences between cancer in mouse models and humans including increased vascular density in several mouse models compared to human tumors.^190^ Also there is concern of poor NP permeation into tumors is due to the high intratumoral pressure upon more advanced tumor development, which can be the result of poor lymphatic drainage in the tumor and eventual buildup of fluids in an acidic and hypoxic environment.^191^ With high intratumoral pressure, the penetration of NPs and even chemotherapeutic agents into the tumor can become limited. Concern regarding EPR‐mediated passive targeting of nanomaterials has led to significant criticism of these technologies for cancer chemotherapy.^192^ Therefore, scientists who develop, characterize, and apply these materials clinically have a responsibility to be rigorous and transparent in their analysis so as to better mitigate concerns and maximize clinical benefit.^193^

Some of the concerns around NPs are exemplified in the path of the company BIND Therapeutics. Accurins, the platform technology behind BIND Therapeutics, consisted of a chemotherapeutic docetaxel‐loaded degradable polylactic‐acid (PLA) NP core with a PEG corona and active targeting through surface ligation of prostate‐specific membrane antigen. Each element of the Accurins contributed a feature to the NP: (1) PLA offered controlled release of a variety of hydrophobic payloads, including chemotherapeutics, that did not have to be modified for encapsulation (Figure 3). (2) PEGylation provided enhanced circulation times and protection against the immune system by providing a stealth layer (Figure 5). (3) Active targeting used a ligand to enrich the NPs at the site of interest (Figure 6) in addition to passive targeting of the tumor via EPR (Figure 7).^194^ BIND Therapeutics was founded in 2006, and AstraZeneca was the first big pharmaceutical company to invest in the company. Over the years, BIND secured several agreements with additional pharmaceutical companies, including Amgen. They raised nearly $70M prior to going public in 2013. A year later, it was announced that Amgen would not be exercising its option on the technology because results were not compelling. Nonetheless, in subsequent years, agreements with Merck and Pfizer were established after positive phase 1 results were reported (NCT01300533). However, in December 2015 they did not meet their outcomes for their Phase II clinical trials for treatment of cervical and head and neck cancers (NCT02479178) resulting in BIND therapeutics filing for bankruptcy in 2016. Assets from the company were bought by Pfizer.^195^, ^196^ While this represents a high‐profile setback for the field of nanomedicine, without difficulty the field cannot advance to the many successes that have come about today. Indeed, critical evaluation of the EPR effect and the use of targeted nanotherapeutics have prompted new insights into fundamental aspects of particle transport into tumors,^197^, ^198^ and more broadly there is consistent growth in understanding the use of NPs as therapeutics. This understanding is highlighted in the wealth of nanotechnology that has made it to clinical trials and has received FDA approval (Table 1).^32^, ^199^

As Table 1 indicates, the time from first report to FDA approval can vary significantly for a given technology, with iron oxide particles taking 140 years from the first report^13^ to a FDA approved product (Feridex),^14^ polymeric particles reaching FDA approval^83^ 13 years after the first published report,^82^ and alum going from discovery of adjuvant activity in animals to human use in only 6 years.^26^, ^27^ Once a product has made it to market and been applied in the clinic, the field continues to move forward. This forward progress is illustrated by the fact that many of the initial products have evolved into second generation formulations or have had market competitors developed. An example is the Smallpox vaccine developed by Jenner,^5^, ^6^ which was replaced by the vaccinia virus in the 1800s to increase availability, and was later discontinued, with the last person receiving the Smallpox vaccine in 1977 because the disease was eradicated.^7^, ^200^ In addition, although Feridex has been discontinued, Resovist (the second FDA approved superparamagnetic iron oxide nanoparticle [SPION] formulation for MRI imaging)^201^ is commercially available. Another example is the discontinuation of Adagen, due to a permanent shortage of the active ingredient,^202^ that paved the way for dozens of PEGylated therapies including Pegasys and Neulasta.^203^, ^204^ These examples illustrate how lessons learned from the shortcomings of one product can lead to development of improved products through the persistence of researchers in the field.

Even after FDA approval, micro and nanotechnology platforms have had their challenges. One of the most well‐known NPs, Doxil, had a shortage from 2012 to 2015 after its sole manufacturing plant was shut down.^205^ The production requirements of the formulations were only managed at Ben Venue Laboratories in Ohio.^206^ To meet the shortage, other PEGylated (Lip‐Dox^206^) and non‐PEGylated (Myoset^207^) liposomes were utilized. Additionally, internationally produced liposomes (Lipodox, Caelyx) were allowed to be imported^208^; however, they were shown to be less effective clinically.^206^ In time, a new manufacturing plant was established and Doxil production was brought back online. The approval of these alternative formulations to fill the need caused by Doxil's absence illustrates the demand for these formulations, underscoring the impact they have on cancer treatment. However, this illustrates the challenge of increased complexity of manufacturing many micro‐ and nanotechnology platforms relative to, for example, small molecule drugs. Indeed, scaling up the fabrication of many micro‐ and nanotechnology platforms and maintaining successful manufacturing at a large scale is a commonly recognized challenge in the translation of these technologies.^209^, ^210^ Fortunately, significant progress has also been made in this area, as exemplified by the manufacturing of COVID‐19 mRNA vaccines. Their manufacturers employed scalable microfluidic technology to generate LNPs that encapsulate mRNA, enabling both the rapid advancement of the vaccines to clinical trials, and the generation of billions of doses to meet worldwide demand.^168^, ^211^

**FIGURE 8:**
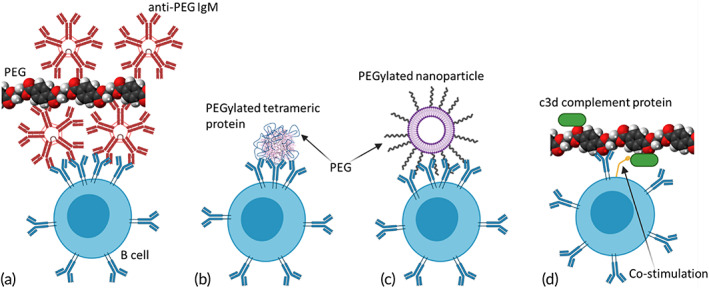
PEG can activate B cells through (a–c) the alternative pathway of B cell activation, wherein surface B cell receptors are clustered, either in response to anti‐PEG IgM binding or the close proximity of multiple repeating PEG chain units. A second way PEG can activate B cells is via (d) complement fixation (particularly c3d protein). Although this type of activation has been shown in other mammals, it has not been illustrated in humans directly.^152^, ^217^, ^218^ Figure not to scale

Other drawbacks of these formulations are in line with those observed for many classes of therapies, including increased formulation production cost, adverse toxicity, and potential off target immune responses.^212^, ^213^ Although Ambisome has been a transformative technology for leishmaniasis, the cost limits the ability of the treatment to be widely administered compared to conventional therapies that are less expensive. For many families in endemic regions, the cost of Ambisome treatment is often greater than their yearly income.^214^ One way that micro‐ and nanotechnologies can reduce cost is through reduction in supportive care^171^ for toxic side‐effects noted with conventional formulations; however, the addition of polymers, lipids, and other materials can create additional toxicity concerns. One example is hand‐foot syndrome where chemotherapy from the formulation leaks into the hands and feet causing redness, swelling, and pain.^215^ Hand‐foot syndrome is observed with Doxil treatment because of the long circulation times of the PEGylated liposome.^216^ In addition to added toxicity concerns, materials added to the active ingredient can result in an unwanted immune response. The injection of PEGylated therapies has been shown to generate anti‐PEG immune responses, particularly IgM responses (Figure 8) and adverse events like anaphylaxis,^217^, ^218^ especially from nanoparticulate PEGylated carriers and PEGylated tetrameric proteins (e.g., PEG‐uricase).^219^ The resulting anti‐PEG antibodies can inhibit the effect of the therapy. However, pretreatment with empty vehicles controls (e.g., Doxebo^220^) has been shown mitigate some of the effects of this response with PEGylated liposome treatments by reducing the concentration of available anti‐PEG antibodies in the blood prior to therapy. Furthermore, prescreening of patients for anti‐PEG responses^152^ can help mitigate some of the observed responses. Similar, but with a much broader and more specific immunological response, the immune response to viral vectors severely limits the ability of a vector to be administered multiples times. One creative way this has been limited is exemplified with the Sputnik V vaccine wherein two different adenoviruses were used (adenovirus 26 [Ad26] and Ad5) because they elicit unique immune responses.^221^, ^222^ Cost, unique toxicity, and aberrant immune responses have all been noted in response to micro‐ and nanotechnologies, which can bring about new challenges in the field.

## 6 LOOKING FORWARD TO THE GROWTH OF MICRO AND NANOTECHNOLOGY

As progress is made in the field, there are opportunities to improve upon products that have already advanced to clinical trials and FDA approval, as well as those that are currently being explored preclinically (Table 1). Moving forward, better handling of aberrant immune responses to the technologies, less expensive production, higher drug loading, and increased personalized and big data approaches to therapy will help to advance these technologies further. Some consideration should be taken by researchers to develop improved and inexpensive bedside diagnostics for anti‐PEG responses as well as new molecules that provide the same outcomes as PEG without the immunological side effects. Similarly, research in improved antigen specific immune reduction for anti‐PEG responses and anti‐viral vector responses could help to advance some of these technologies. Another area of growth for these technologies is additional scalable processes as well as inexpensive production methods, particularly for modular platform technologies like LNPs. When the next pandemic or infectious disease outbreak occurs (e.g., Ebola), rapid onsite generation of new therapies and vaccines would be transformative in resource limited settings. Moreover, increased loading percentages of cargo within existing and new technologies are needed. As exemplified with ART, drug nanocrystals could be used because a majority of the technology was the drug compound (~80%), as opposed to liposomes or polymeric microparticles where loading is often 30% or less of the overall formulation weight. With ART, in particular, the amount of drug needed for over a week's treatment is nearly infeasible for the mass of formulation that can be injected into a human. For example, long acting rilpivirine (TMC278 LA) is injected at two sites for a total of 1200 mg of drug formulation, each in 1 ml injection (NCT02165202). If drug loading were less than the 80% noted, then the drug could not be delivered at an effective dose. Furthermore, rilpivirine has one of the lowest doses required of all ARTs (25 mg daily), which in some cases is orders of magnitude less mass than needed for other common ARTs (e.g., emtricitabine 200 mg/day, lamivudine 300 mg/day, abacavir 600 mg/day). This makes most ARTs less feasible to deliver in a long‐acting injectable formulation at therapeutic levels despite the bioavailability and metabolism benefit of injectable delivery compared to orally delivered compounds (e.g., bioavailability is 100% for intravenous compounds and avoids first‐pass metabolism compared to oral). By increasing the drug loading of existing and emerging technologies, additional cargoes and dosing levels required to mitigate resistance can be more aptly delivered. Finally, as the field moves forward, tuning these platform technologies to have a more personalized approach will improve outcomes. This personalization requires greater expertise in machine learning and big data management. With critical information gleaned from initiatives like the Cancer Genome Atlas project, disease genotyping, and pathogen sequencing, researchers are generating ever‐increasing amounts of data, and scientists/engineers and platforms that can digest and implement this data into useful technologies that meet the individual needs of the patient are needed. Training in data science with genomics and other omics is needed to develop these optimized drug delivery platforms. These advancements could help push the fields of new polymers, lipids (particularly ionizable lipids), and nanoparticles further into a “space‐race” of their own, driving research further forward for cancer, infectious diseases, and chronic diseases such as cardiovascular conditions. With further advancement, micro‐ and nanotechnologies can be pushed beyond what was sparked by the National Nanotechnology Initiative in 2000 to generate more exciting and transformative platforms for the benefit of human health.

## AUTHOR CONTRIBUTIONS

**Rebeca T. Stiepel:** Writing – review and editing (equal). **Eliza Duggan:** Validation (equal); writing – original draft (equal); writing – review and editing (equal). **Cole J. Batty:** Writing – original draft (equal); writing – review and editing (equal). **Kristy M. Ainslie:** Resources (equal); supervision (equal); writing – original draft (equal); writing – review and editing (equal).

### PEER REVIEW

The peer review history for this article is available at https://publons.com/publon/10.1002/btm2.10421.

## Data Availability

Data sharing is not applicable to this article as no new data were created or analyzed in this study.
